# Supplementary figures and images for: Apoptosis mapping in space and time of 3D tumor ecosystems reveals transmissibility of cytotoxic cancer death
Source: PLoS Comput Biol. 2021 Mar 30;17(3):e1008870. doi: 10.1371/journal.pcbi.1008870 (PMC8034728; doi:10.1371/journal.pcbi.1008870)

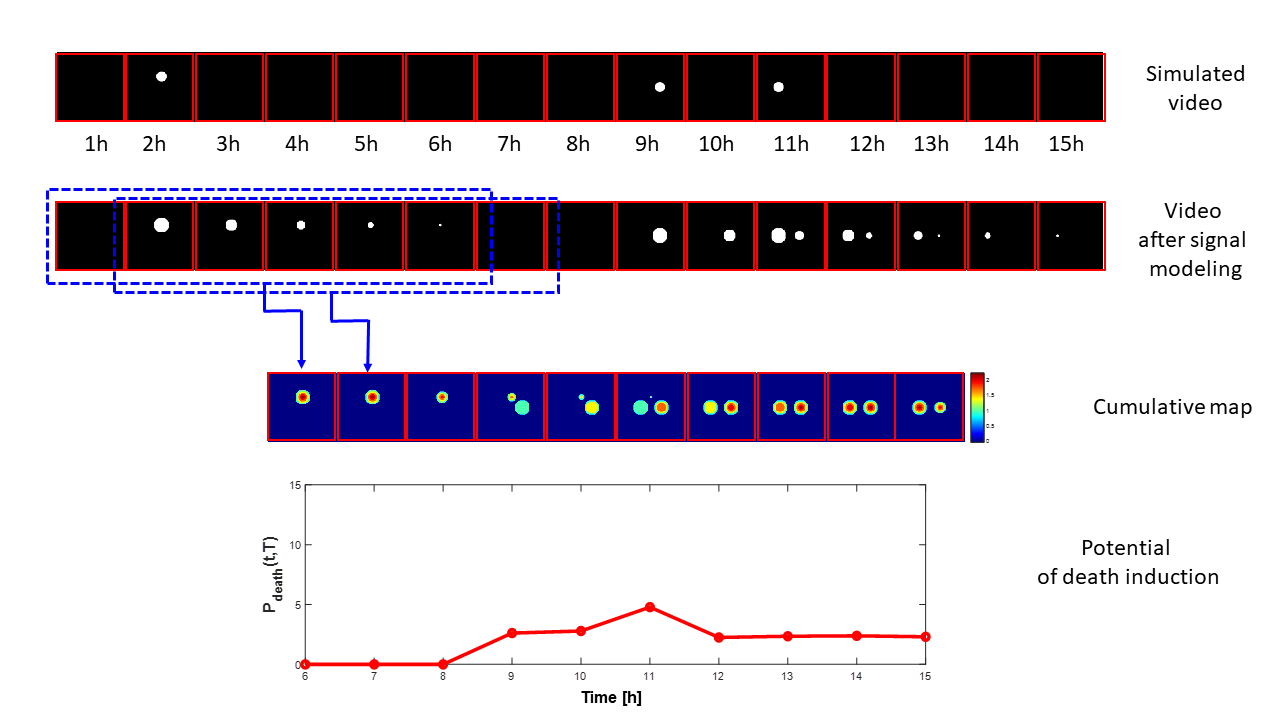

Supplement: S1 Fig — Three simulated deaths occur at 2 h, 9 h, and 11 h (first video sequence). The death signals are modeled by the construction of a signal wake (second video sequence), the duration of which depends on the dimension of the original death region (first video sequence). Then, a cumulative map MC(x,y,t,T˜) is constructed by combining both spatial and temporal death influence (third video sequence) using T˜=6. Finally, Pdeath is computed over time for the entire image area (bottom graph). Until t = 8 h, there is only one death, so there is no induction phenomenon. An additional death occurs at t = 9 h thus producing an induction phenomenon and an increase in potential. A third death occurs at t = 11 h thus producing a further increase in the potential value. Potential is also influenced by the absolute value of the map MC and by the distances of the different zones of death. From t = 12 h there is no more memory of the first death, hence only the last two death zones remain whose distance is larger than that of the two death zones involved in t = 9 h and 11 h, thus causing a decrease in potential. (PNG) [file pcbi.1008870.s001.png]

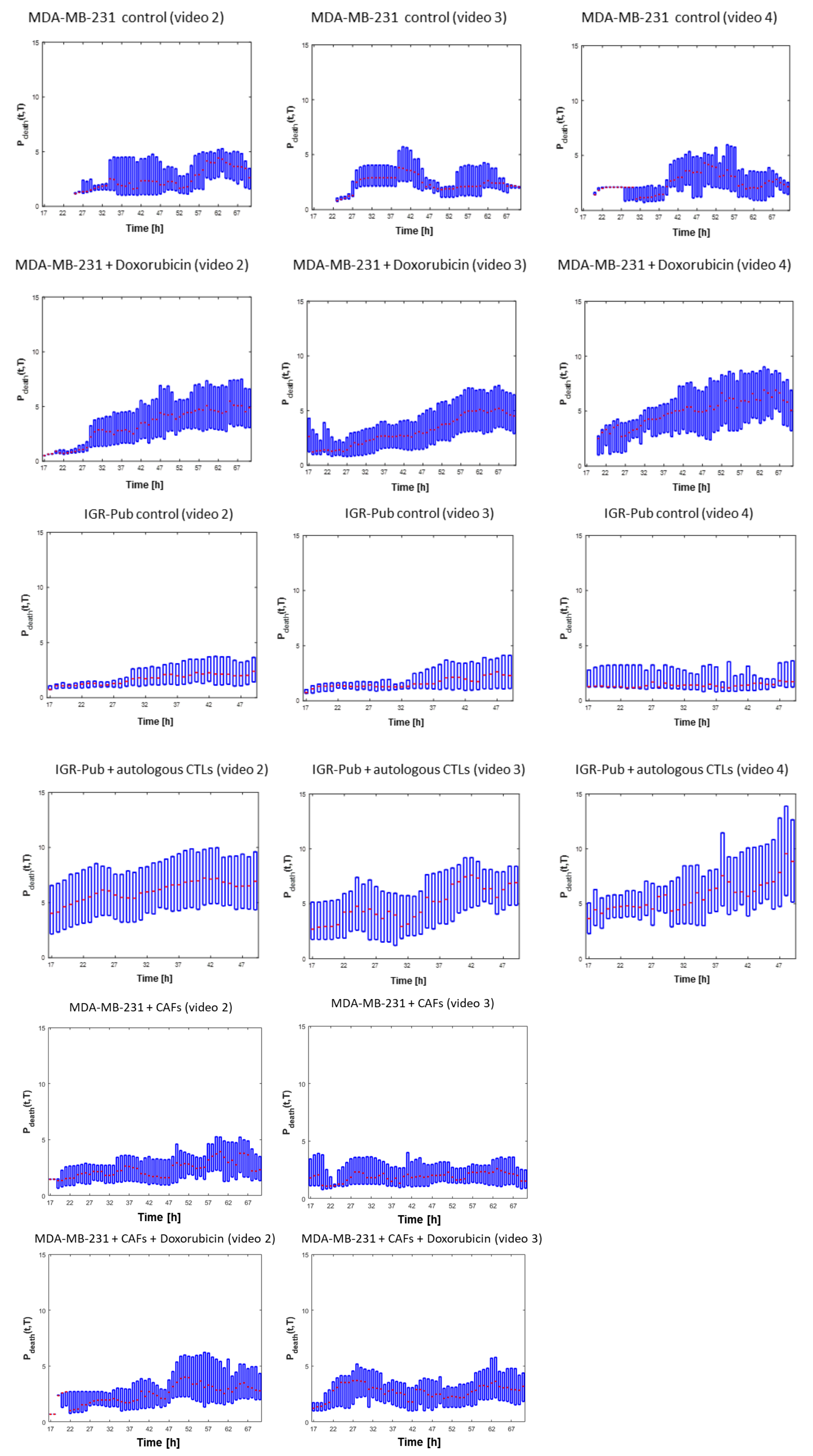

Supplement: S2 Fig — (PNG) [file pcbi.1008870.s002.png]

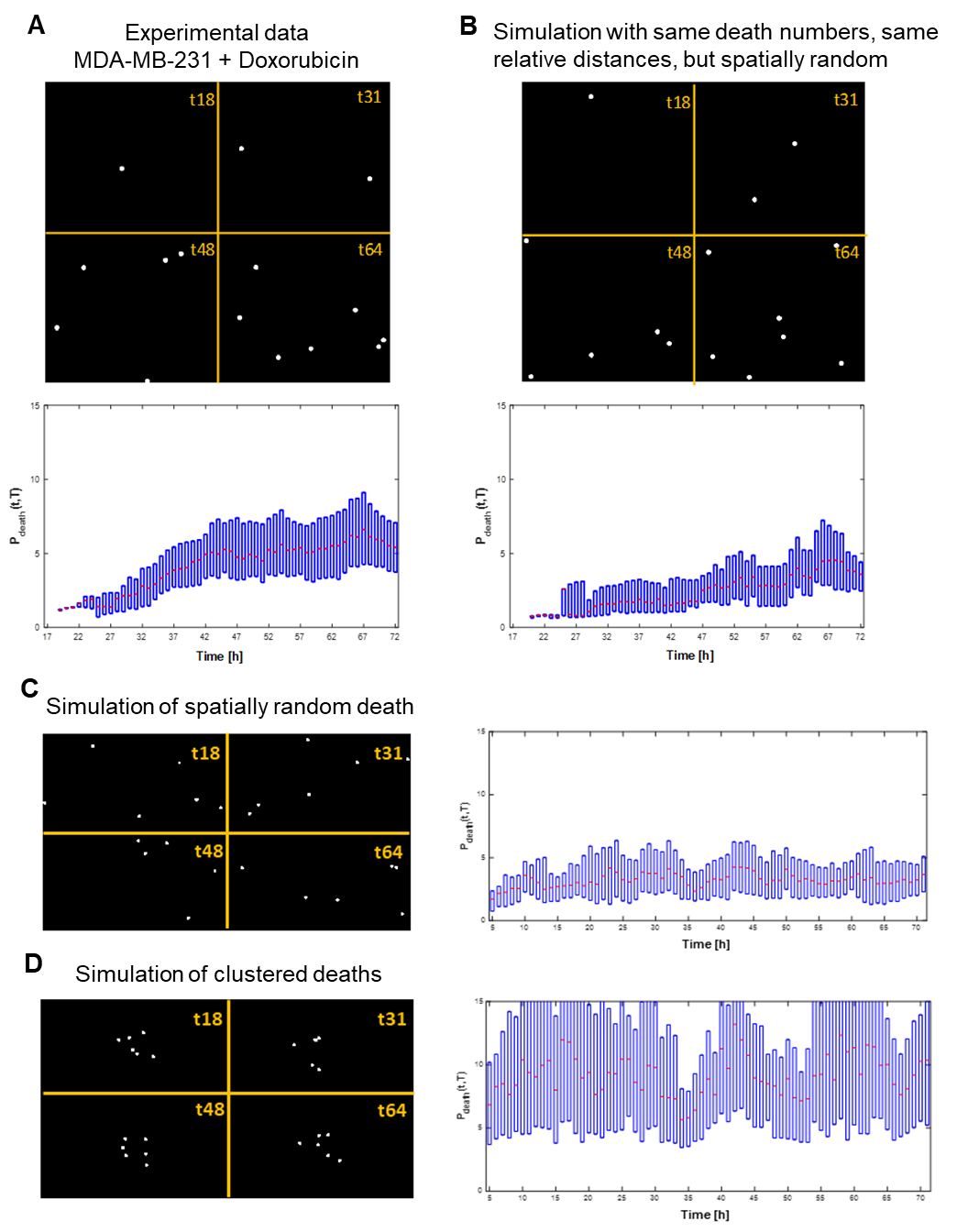

Supplement: S3 Fig — A. Experimental data showing the spatial localization of death events at different time points (above), and the corresponding Pdeath measurements (below) on a video of MDA-MB-231 cells treated with 1 μM doxorubicin (the same reported in Fig 6A). B. Simulation of a video with the same death events as in A, but with a spatially random distribution, maintaining approximately the relative object distances. Note that the corresponding Pdeath measurements are increasing much less than in A, indicating that the Pdeath increase does not simply result from the increase of death numbers over time, but it depends also on death positions. C. Simulation of a video with a constant number of death events with a spatially random distribution. Note that the corresponding Pdeath measurements are constant over time. D. Simulation of a video with a constant number of death events with a clustered distribution. Note that the corresponding Pdeath measurements are constant over time, but higher than in C. (PNG) [file pcbi.1008870.s003.png]

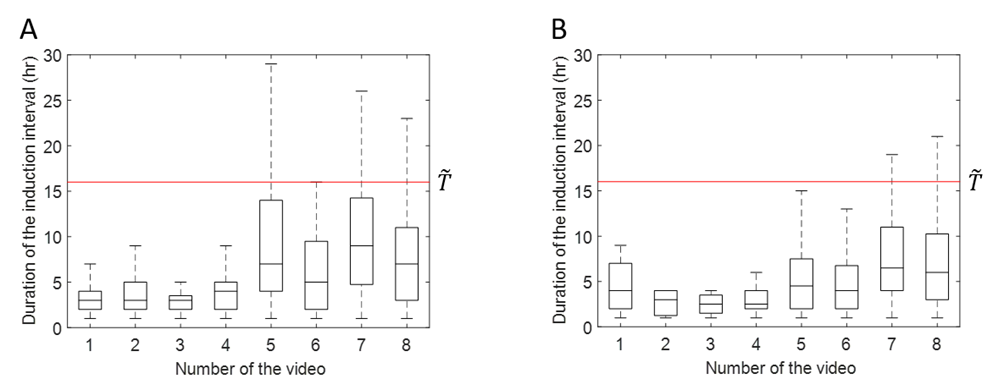

Supplement: S4 Fig — T˜ is the time window over which the aggregation of deaths and their wake were computed by means of the definition of the cumulative map MC (Eq (9)). The induction intervals, defined as the duration of the chain of death, were computed for each cell, from 16 videos from 2 experiments, one experiment with the lung cancer cell line IGR-Pub (A) and one experiment with the breast cancer cell line MDA-MB-231 (B). The distributions of induction show that vast majority of induction intervals is below 16 h, meaning that T˜=16 h is an optimal choice. (PNG) [file pcbi.1008870.s004.png]

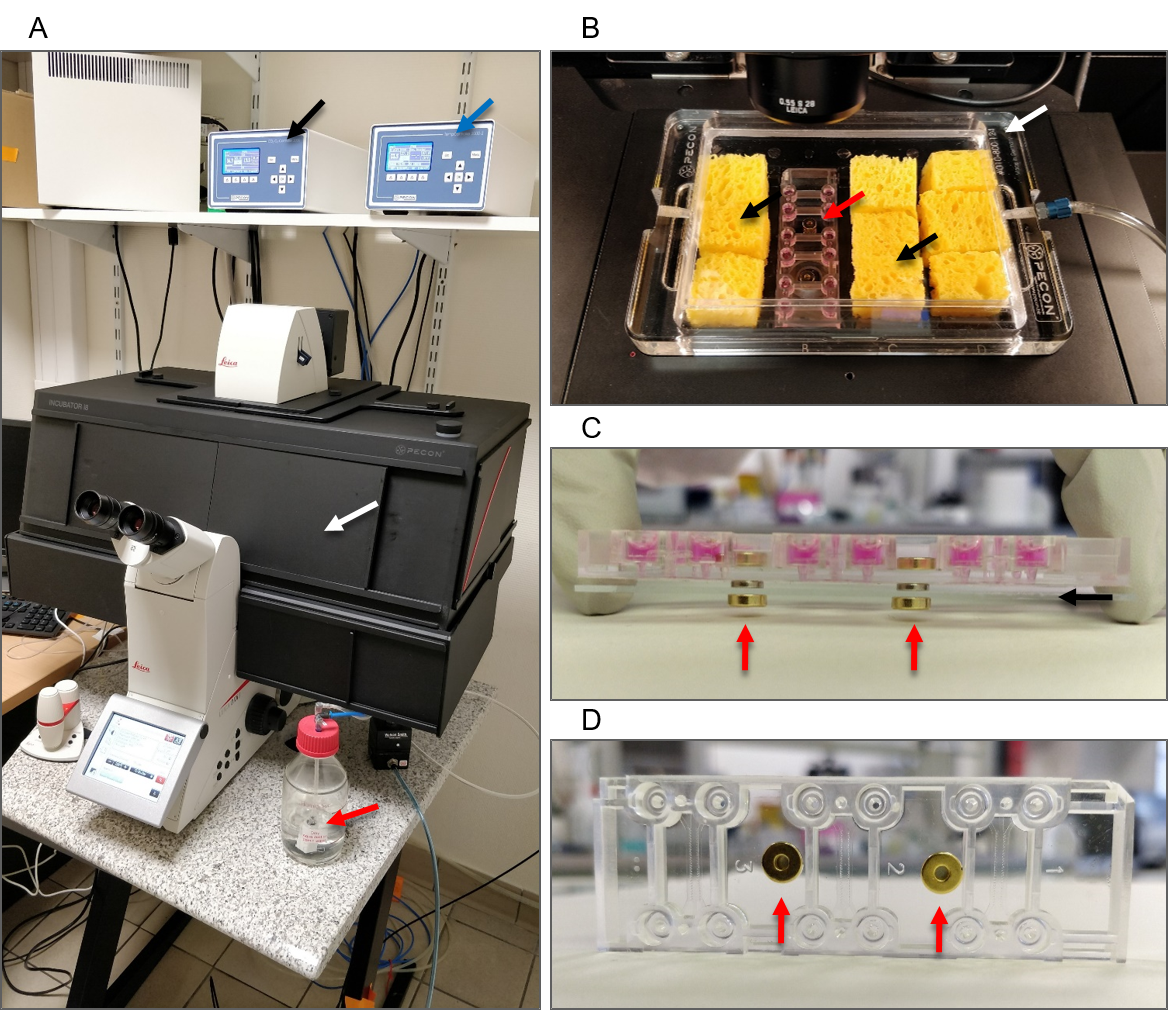

Supplement: S5 Fig — A. Global view of the Leica DMi8 used to perform the live imaging experiments. The white arrow points at the heating black chamber, in which is maintained a temperature of 37°C. The temperature is set and maintained by a temperature controller (blue arrow). The CO2 controller (black arrow) mixes CO2 with atmospheric air. Through a tubing system, the air with the controlled CO2 at 5%, after passing through a bottle half filled with water for humidification (red arrow), is injected in the microscope chamber. B. View of the chip placed in the microscope chamber. The white arrow indicates the lid of the chamber in which is injected the humidified air with CO2 at 5%. The red arrow points at the chip to be imaged. Humidified sponges contribute to humidify the chamber and to minimize micro-evaporation phenomena (black arrows). C. Picture from the side of the chip filled with medium. At each ‘anchor’ point, three magnets (red arrows) are used to lift and attach the chip to the glass slide (black arrow). D. Top view of an empty chip with magnets. Two piles composed of three magnets (red arrows) are applied in the central part of the chip and glass slide. (PNG) [file pcbi.1008870.s005.png]
