# Supplementary material for: Apoptosis mapping in space and time of 3D tumor ecosystems reveals transmissibility of cytotoxic cancer death
Source: PLoS Comput Biol. 2021 Mar 30;17(3):e1008870. doi: 10.1371/journal.pcbi.1008870 (PMC8034728; doi:10.1371/journal.pcbi.1008870)
Supplement: Attachment — Submitted filename: Responses to reviewer v10.pdf [file pcbi.1008870.s013.pdf]

Answer to Reviewer #1

We thank Reviewer #1 for acknowledging the interest of our work to the readership of PLOS Computational Biology. We extensively revised the manuscript addressing all the points that were raised.

Page 4, under application to quantify chemotherapy-mediated cytotoxicity: the authors observed apoptosis rate fluctuated around 5% under control condition (Figure 2C), especially around 20-50 h, is that a normal apoptosis rate the author would expect?

The values of basal apoptosis rate reported in literature for MDA-MB-231 cells, using flow cytometry with reagents such as Annexin V and propidium iodide, are approximatively 2-5 % apoptotic cells per day [1,2]. Our average measurements for MDA-MB-231 control fluctuate in the range of 1 to 6 % apoptotic cells per 10 hrs (Figure 2C). Considering the differences in experimental setting (2D vs 3D) and in detection methods (flow cytometry vs caspase live reporter), we can conclude that the observed basal apoptosis rates are as expected.

1. Ding L, Chu M, Jiao Y, Hao Q, Xiao P, Li H, et al. TFDP3 regulates the apoptosis and autophagy in breast cancer cell line MDA-MB-231. PLOS ONE. 2018;13: e0203833. doi:10.1371/journal.pone.0203833
2. Varghese E, Samuel SM, Varghese S, Cheema S, Mamtani R, Büsselberg D. Triptolide Decreases Cell Proliferation and Induces Cell Death in Triple Negative MDA-MB-231 Breast Cancer Cells. Biomolecules. 2018;8. doi:10.3390/biom8040163

Page 5, under application to quantify T-cell mediated cytotoxicity: There seems to have some discrepancy between figure 3C and 4. In Figure 3C, coculture of IGR-Pub and CTL gave apoptosis rate around 15% at 20-30 h, while in Figure 4, the rate for interval 20-28 h is already over 40% (24% for 20-24 h+19% for 24-28 h). Seems like the authors are using the exact same set of videos for quantification, can the authors provide some explanation about this discrepancy?

Data of Figure 3 and Figure 4 are generated from two separate experiments. It is now specified in the revised manuscript (page 5, line 189).
The discrepancy between the two sets of measurements is explained by three reasons. First, the cytotoxic activity of the T-cell clone depends on its activation status; when cytotoxicity is performed early after reactivation of T lymphocytes with irradiated autologous tumor cells (feeder cells) and lymphoblastoid B cells, specific lysis of target cells is stronger. Second, even minimal variations of the ratio between effector and target cells can be source of inter-experiment variability, due to the marked cytotoxic activity of the effector T cells. Third, the apoptosis in 10h-time-interval (Figure 3) is not mathematically equal to 2.5 fold the apoptosis in 4h-time-interval (Figure 4) since the percentages refer to the time intervals, not to the entire experimental time; the percentages are calculated as the sum of the deaths registered in a given time interval (4 or 10 hours), over the initial number of living cells, at the beginning of this specific time interval.

Page 5, under application to quantify T-cell mediated cytotoxicity: the authors indicated "after

2 days on chip the T cell viability was reduced (around 90 %)". It would be better if the authors could provide more details in how to measure the viability for T-cells, either in this paragraph or in method.

Since the fluorescent apoptosis reporter is added to culture medium, all cells dying by apoptosis will become green. We then manually counted the T cells that became green during the experiment time. We added a sentence in this paragraph to explicit this point (page 5, line 196). Moreover, we performed additional manual counts to specify a more precise value for T cell apoptosis over 2 days: 19.7 ± 3.1 % (around 80 % viability), values added to the same paragraph.

Page 5, under cancer-associated fibroblasts promote chemo-resistance: the authors need to provide statistical analysis for the four conditions in Figure 5, especially the conditions +dox/-CAF and +dox/+CAF. The curves looked different, however the difference might not be significant due to the error (curves were plotted using SEM, not SD). The same applied for Figure 4A and 4B.

Reviewer is correct. We now provide statistical analyses for Figure 4 and Figure 5. The statistical p values now appear inside the figures. The 3 measurements are technical replicates (different view fields from the same experiment). In order to compare the different conditions, regardless of the time variable, the measurements for each condition were assembled (n=36 for Figure 4, n=51 for Figure 5). We first performed a Shapiro-Wilk normality test. When the conditions passed the normality test, we applied an unpaired t test. Where else, we performed the non-parametric Mann-Whitney test. These statistical points are specified in the legend texts.
We added new sentences in the Materials and Methods section to better describe the statistical analyses (page 13, line 602).

Page 5, under spatiotemporal analysis of cytotoxicity death: it would be clearer to define "nearby cells" here, eg. how close the dead event would be considered as a nearby event.

We added a sentence to explicit this point (page 5, line 222): "Of note, since the window size for the calculation of the Pdeath is 283 μm x 283 μm, a nearby cell would be a cell within this window".

Is the definition "one third of the estimated average cell radius" applied to all the quantification?

Yes, it does.

Would cell density significantly affect the outcome?

At constant condition (same cell type, same drug treatment), assuming spatially random death, the density of cells should be proportional to the density of death events. The density of death events strongly affects Pdeath, as possible to extrapolate from the formula (11), which takes into account both the number of deaths $|S(t)|$ and their relative distances $\bar{d}(s_i(t), s_j(t))$. However, Pdeath increase does not simply result from the increase of death numbers over

time, but it depends also on the death positions, as shown by the simulations reported in Supplementary Figure 3.

Also it would be interesting to see the result for MDA-MB-231 ±dox/+CAF in main text or supplementary info. Based on the result discussed in previous section, CAF impaired doxorubicin dependent apoptosis and therefore the Pdeath would be expected to be similar as natural death.

We thank Reviewer for this interesting idea. We run the Pdeath analysis on the videos with CAFs. The results are now shown in a new Figure 6C and new panels on Supplementary Figure 2.
As correctly predicted by Reviewer, Pdeath is not affected by the presence of doxorubicin when CAFs are present. A sentence has been added to the Result section to comment this new finding (page 3, line 241).

Page 6, under the discussion for STAMP method, paragraph 2: Navg(t, TLAG), "N" should be italic

Done.

Page 7, under the discussion for biological implications, paragraph 1: "in vitro" should be italic. "The CTL clone (P62) used is this work kills autologous cells (IGR-Pub)", should be "used in this work"

Done.

Page 8, methods for cell cultures: for cell line authentication, the authors presumably refer to STR profiling instead of SRT? Maybe spell out this abbreviation.

Done.

Page 9, methods for live cell imaging: it would be better if the author could provide more details in setting up the time-lapse image acquisition, eg, wavelength, filter, exposure, etc.

We added new sentences in the Materials and Methods section to better describe the microscope setup (page 9, line 412) and all the requested information can now be found there.

And seems like the way to position the AIM-Biotech devices is a key step in experimental setting. It would be helpful if the authors could provide some photos in supplementary info about this setting, which will enable easy application of this method by other audience.

We added a new Supplementary Figure 5 with photos depicting the right position of the AIM-Biotech devices on the microscope stage, within the 37°C-heated CO_2-controlled microscope chamber.

Page 12, the authors are using Mann-Whitney-Wilcoxon nonparametric test through the entire manuscript, however, the sample size (n=3) is too small to achieve any statistical meaning. As it is stated on the GraphPad's website (https://www.graphpad.com/guides/prism/8/statistics/how_the_mann-whitney_test_works.htm), "If you have small samples, the Mann-Whitney test has little power. In fact, if the total sample size is seven or less, the Mann-Whitney test will always give a P value greater than 0.05 no matter how much the groups differ."
Also, in many figures, for example Figure 2C, it seems clear that MDA-MB-231 control 0-10, MDA-MB-231 doxorubicin 0-10/30-40 are different, however, due to the small sample size, there is no significant difference achieved. And therefore, the statement on page 4 "we never found statistically significant differences between automated and manual counting, indicating that the algorithm is validated with respect to a standard human-controlled quantification method" is not appropriate. The authors need to increase the sample size to support their statement. I would envision there would be significant difference between manual counts and automatic counts, can the author provide more details about why that would occur, and is there any improvement can be made. In addition, can the authors explain why they chose Mann-Whitney-Wilcoxon test instead of t test?

The Reviewer is totally right; we are very grateful for pointing to this mistake: the Mann-Whitney test is not appropriate when n=3.
We did increase the measurements for the automatic quantifications, which are now providing every hour in the revised versions of Figure 2C and Figure 3C.
Manual and automated counting reach numbers in very similar ranges. Indeed, as pointed by the Reviewer, some discrepancies are evident, for example for the first time points (t=10h) in Figure 2C, where manual measurements are slightly higher than automatic ones. Even if the numbers are not identical, the curve trends are very similar. This is what matters, since results of interest are more often evolutions of such numbers (according to time, to dose of drugs…), rather than the absolute values
Importantly, we could reach a much higher (10-fold) time resolution for automated measurements (every hour) than for manual measurements (every 10 hours), because performing manual counting every hour becomes an extremely annoying and time-consuming burden.
We thus removed the inappropriate statement and we replaced it with a more rigorous interpretation, thanks to reviewer's remark, to support the conclusion that our automated counting actually is a *bona fide* method to measure apoptosis rate (page 4, line 161).

And for the replicates, are they technical replicates (different views) or biological replicates (different samples)? I would recommend using biological replicates to achieve solid result.

We better explained, in the legend texts, that the measurements were technical replicates (different view fields from the same experiment). We performed several times the experiments, obtaining qualitatively the same biological conclusions. For example, data reported in Figure 2 and Figure 4 come from independent experiments. The focus of Figures 2, 3, 4 and 5 is not on the biological results, which are expected, but on the compatibility of the automated method with co-cultures, with T-cell or CAFs. So we think that our conclusions reported here are sound.

Page 19, figure 2B, please define blue arrow. Presumably the authors are indicating CTL cells?

Done, the comment refers to Figure 3B, we guess.

Also, I don't find the info related to deposit of this software. It will be helpful if the authors could provide a link/user manual to guide the audience getting access to this tool.

The STAMP software is open source, as now clearly mentioned in the abstract (page 2, line 46). The link where the software is deposited, together with a tutorial for its use, is indicated in the Materials and Methods section (page 10, line 429).
https://cloudstore.bee.uniroma2.it/index.php/s/LEpHYTsPnDj4Ajt
Password: STAMP2021

Answer to Reviewer #2

We thank Reviewer #2 for recommending publication of the manuscript with no revisions.
