# Supplementary material for: Apoptosis mapping in space and time of 3D tumor ecosystems reveals transmissibility of cytotoxic cancer death
Source: PLoS Comput Biol. 2021 Mar 30;17(3):e1008870. doi: 10.1371/journal.pcbi.1008870 (PMC8034728; doi:10.1371/journal.pcbi.1008870)
Supplement: Attachment — Submitted filename: Response_2_to_Reviewer_v2.docx [file pcbi.1008870.s014.docx]

Response to Reviewer #1

We thank Reviewer for recommending publication of our work. Here, our answers to the last few minor points.

***Q:*** *For T cell quantification, seems like the method is via manually counting. As this manuscript is all about developing an algorithm to simplify video analysis, is it possible to establish an automatic way to streamline the process? Or what is the difficulty in developing such method? Please discuss more in the manuscript.*

**R:** In this work, the main attention was devoted to the death of cancer cells, because in cancer treatment the ultimate goal is to kill the cancer cells. For this task, we implemented automatic cancer cell localization and tracking procedures in the red channel, with a low temporal resolution (1 h) which was perfectly appropriate for our goal. Regarding the T cells, that are fast moving, the 1h time resolution is not appropriate for their tracking (see also our recent study of the required frame rate for T-cell investigation, Comes et al., 2019, Sci-Rep). T-cell tracking would require a temporal resolution <1 min, which is feasible, the only limitation being the amount of data to be stored and handled. Indeed, with the appropriate adjustments, the STAMP method can be used to measure the death rate of any cell type of the tumor microenvironment. We added a sentence in the Discussion (page 6, lines 270-276) to specify this point:

“In principle, the STAMP method could be used to measure the death of other cells types within ToC, such as immune cells or fibroblasts, by selectively pre-staining one of these populations. However, some adjustments will be required. First, the time acquisition rate needs to be adapted to the motility of the specific cell type; for example, T-cell tracking would require a temporal resolution much higher than cancer cells, with time intervals less than 1 min (Comes et al., 2019, Sci-Rep). Second, the STAMP code needs to be adapted to different cell morphologies; for example, the cell radius parameter must be revised.”

***Q:*** *In terms of the definition about “nearby cells”, I am still a little confused, as I thought the parameter “r” restricts the event of nearby cells (death wake). While as the authors wrote in the response, it is included all the cells within the detection window? Can the authors provide more explanation towards this question?*

**R:** The role of “r” is to construct the death wake around each dead cell along time. Conversely, the size of the windows, here 283 μm x 283 μm, is used to restrict the computation of the Pdeath on the death events occurring in a smaller portion of the entire view field.

To avoid any further confusion between “r” for death wake construction and detection window for Pdeath calculation, we added a new sentence in M&M (pages 14, lines 611-614) in order to better specify the significance of the detection window:

“In the computation of the Pdeath, Eq. (11) is repeatedly applied to a sliding detection window of dimension 283 μm x 283 μm to cover the entire view field. In this way, not only the death signal is spatially confined, but also the statistical relevance of the calculus is increased at each time point. We define as ‘nearby cells’ those falling in this 283 μm x 283 μm window.”

***Q:*** *Also for the discrepancy between manual and automated counting, to me there is significant difference between this two groups at certain time point (in figure 2c). Please perform statistical test and specify accordingly on the figure. Looks like the discrepancy is more likely to occur at early time point, is there any reason why automated counting has lower readout at early time point? Why the algorithm failed to pick apoptotic cells which could be easily identified via manual counting? Please provide more discussion about this discrepancy.*

We performed statistical tests in Figures 2C, and also Figures 3C for consistency. The test method (non-parametric Mann-Whitney) and results (not significant) are now indicated in the modified legends.

We used the non-parametric Mann-Whitney test, and not a t-test, because the data did not pass the normality test. This is expected since the variable under test represents a counting variable. As underlined in the reference metrological documents (Evaluation of measurement data — Supplement 1 to the “Guide to the expression of uncertainty in measurement” — Propagation of distributions using a Monte Carlo method SUPPLEMENT 1, JCGM 101:2008, <https://www.bipm.org/utils/common/documents/jcgm/JCGM_101_2008_E.pdf>, section 6.4.11), variables whose domain is bounded below at zero (e.g., counting variables), and whose value is close to 0 cannot be represented by a symmetric Gaussian distribution.

Even though the statistical analysis indicated that there are no significant differences between the manual and automated counting when the time variable is not taken into account, in some cases manual counting resulted higher than automated counting (e.g. early time points with MDA-MB-231 cells). We added a new sentence (page 4, lines 163-168) to comment these minor discrepancies:

“We observed that the algorithm had a tendency to underestimate the apoptotic cells at early time-points. This might be due to the fact that the green channel threshold, that is used to detect apoptotic events (Figure 1), is automatically determined over the 72 hours. Early, weak signals may be lost because under the threshold. Conversely, a human operator cannot discriminate between weak and strong green emissions and therefore may assign apoptotic events to any green emission increase.”
